# Maternal collapse in pregnancy: evolving aetiology and healthcare system response

**DOI:** 10.1016/j.crwh.2025.e00750

**Published:** 2025-09-18

**Authors:** Danny Tucker, Melissa Freestun

**Affiliations:** a James Cook University, Australia; b School of Healthcare Sciences, James Cook University, Australia

**Keywords:** Pregnancy, Collapse, Critical care, Obstetric medicine, Cardiac arrest

Maternal collapse demands immediate recognition, pregnancy-specific clinical protocols, and decisive consideration of resuscitative hysterotomy if the initial response proves inadequate. Maternal and neonatal survival is contingent upon skilled clinicians and readily available equipment. Clinical responses and system preparedness are required to adapt to the local causes of maternal decompensation and collapse [1,2].

The aetiology of maternal collapse varies across the world. Haemorrhage remains the leading cause of maternal death worldwide, followed by indirect causes and hypertensive disorders. However, the relative proportions differ markedly by geographical region: haemorrhage predominates in sub-Saharan Africa and parts of Western Asia and Northern Africa, whilst hypertensive disorders represent the leading cause in Latin America and the Caribbean. Effective obstetric emergency capabilities are required across all healthcare settings [3].

Cardiovascular disease is a frequent cause of pregnancy-related death in high-income countries, and many deaths occur following discharge from the hospital. Between 2018 and 2022, the United States reported a 27.7 % increase in pregnancy-related mortality, with cardiovascular disease the leading cause. Additionally, there are marked racial, ethnic and geographical disparities. The puerperium represents a potential blind spot for healthcare systems traditionally focused on antenatal and intrapartum care [4].

Multiple converging factors explain this epidemiological transition, including a higher prevalence of advanced maternal age and greater cardiometabolic burden. Improved survival rates for conditions such as congenital heart disease mean that more women with complex cardiac anatomy reach reproductive age and fall pregnant. These demographic shifts mean that maternal collapse may more commonly encompass primary cardiac rather than traditional obstetric causes. Proactive risk stratification through multidisciplinary pregnancy heart teams, using established frameworks such as the modified WHO classification system, has become essential for both prevention and emergency preparedness [5,6].

Effective cardiopulmonary resuscitation adapted for pregnancy remains the foundation response. Key priorities include immediate manual left uterine displacement to relieve aortocaval compression, prompt airway management by the most experienced available clinician, and systematic evaluation of reversible causes whilst maintaining effective chest compressions. Where the uterine fundus is at or above the umbilicus (approximately ≥20 weeks) and cardiac arrest persists beyond the initial minutes, teams should proceed to resuscitative hysterotomy without adherence to arbitrary time thresholds. The traditional four-minute rule is a clinical aide-memoire rather than a specific pathophysiological time threshold. Each minute of delay reduces the likelihood of achieving return of spontaneous circulation and, where applicable, fetal survival [1,2].

Maternal collapse is frequently preceded by detectable clinical deterioration. Maternity observation scoring systems, such as the Modified Early Obstetric Warning Score (MEOWS), can facilitate prompt response to the deteriorating patient. These systems have moderate reliability to predict admission to intensive care facilities and maternal morbidity. Successful implementation requires concurrent auditing of escalation processes and clinical response patterns to ensure system effectiveness [7].

Since maternal cardiac arrest is infrequent, institutional preparedness requires systematic preparation. Regular multiprofessional in-situ simulation training that rehearses obstetric cardiopulmonary resuscitation and resuscitative hysterotomy, clarifies team roles, and practises logistical coordination has been associated with measurable process improvements. Whilst evidence suggests benefits for selected patient outcomes, effect sizes vary considerably and training methodologies significantly influence effectiveness [8].

Cardiovascular-related maternal deaths, including late mortality, disproportionately burden marginalised populations. Postpartum care pathways need to integrate cardiology review, structured handover to primary care and patient education. These are likely to be equally important as intrapartum emergency protocols. The optimal outcome remains preventing collapse through proactive risk modification and timely clinical care [4].

Healthcare systems must understand the local epidemiology of maternal collapse and design services accordingly. This includes clinical simulation in maternity and emergency department settings. Implementation of reliable maternity early warning systems with robust escalation pathways should be standard practice. Finally, structured postnatal cardio-obstetric follow-up for women with known or suspected cardiac disease is required. As the underlying aetiology of maternal collapse changes, healthcare systems must adapt too.

## Contributors

Danny Tucker contributed to drafting the manuscript and revising the article critically for important intellectual content.

Melissa Freestun contributed to drafting the manuscript, undertaking the literature review and revising the article critically for important intellectual content.

Both authors approved the final submitted manuscript.

## Provenance and peer review

This editorial was commissioned and not externally peer reviewed. Danny Tucker, an editorial board member of *Case Reports in Women's Health*, was not involved in editorial consideration of the manuscript and was blinded to the process.

## Declaration of generative AI and AI-assisted technologies in the writing process

During the preparation of this work the authors used Grammarly to check spelling and grammar. After using Grammarly, the authors reviewed and edited the content as needed and take full responsibility for the content of the published article.

## Funding

No funding from an external source supported the publication of this editorial.

## Declaration of competing interest

The authors declare that they have no competing interest regarding the publication of this editorial.
